# The Role of Lifestyle Behaviors on 20-Year Cognitive Decline

**DOI:** 10.1155/2012/304014

**Published:** 2012-09-04

**Authors:** D. Cadar, H. Pikhart, G. Mishra, A. Stephen, D. Kuh, M. Richards

**Affiliations:** ^1^MRC Unit for Lifelong Health and Ageing, London WC1B 5JU, UK; ^2^Faculty of Population Health Sciences, University College London, London WC1E 6BT, UK; ^3^School of Population Health, University of Queensland, Herston, QLD 4006, Australia; ^4^MRC Human Nutrition Research, Elsie Widdowson Laboratory, 120 Fulbourn Road, Cambridge CB1 9NL, UK

## Abstract

This study examined the association between smoking, physical activity and dietary choice at 36 and 43 years, and change in these lifestyle behaviors between these ages, and decline in verbal memory and visual search speed between 43 and 60–64 years in 1018 participants from MRC National Survey of Health and Development (NSHD, the British 1946 birth cohort). ANCOVA models were adjusted for sex, social class of origin, childhood cognition, educational attainment, adult social class, and depression; then the lifestyle behaviors were additionally mutually adjusted. Results showed that healthy dietary choice and physical activity were associated, respectively, with slower memory and visual search speed decline over 20 years, with evidence that increasing physical activity was important. Adopting positive health behaviors from early midlife may be beneficial in reducing the rate of cognitive decline and ultimately reducing the risk of dementia.

## 1. Introduction

With an increase in the ageing population, the number of older people affected by cognitive decline and dementia is continually rising, causing a major public health impact on individuals and governments around the world [1]. Despite major progress in understanding the neurobiology of cognitive impairment and dementia, there are still no clear determinants and complete causal models available for explaining risks for this condition [2]. Substantial evidence suggests that certain lifestyle behaviors (particularly smoking, sedentary lifestyle, and poor dietary choices) predict faster cognitive decline [3–5], and higher risk of dementia [6, 7], while physical, mental and social leisure activities are found to be protective [8, 9]. A study of London civil servants [10] highlights that the number and duration of unhealthy behaviors are associated with subsequent cognitive function in later life. Similar findings from the Suwon Longitudinal Aging Study (SLAS) showed that a combination of multiple positive lifestyle behaviors (such as nonsmoking, vegetable consumption, and social activity) was associated with higher cognitive ability [11]. However, since these behaviors tend to cluster [12, 13], the extent to which apparent effects of one behavior are attributable to (i.e., confounded by) another is uncertain.

In addition, relatively little is known about the longitudinal effects of these behaviors on cognitive decline; yet associations among multiple health behaviors place the emphasis on longitudinal studies, since patterns of behaviors tend to develop over decades, with implications for targeted interventions to change the aggregate public health risk [14]. The life course approach to age-related diseases [15, 16] provides an important opportunity to identify the nature and timing of different environmental contributions to neuronal damage and the risk of dementia across life [17]. The present study therefore focuses on behavioral risk in early midlife, at a stage of the life course when people are still more likely to have control over modifiable risk and protective factors for cognitive decline than in later life.

Although clinically significant cognitive decline and the onset of dementia occur in older age (65+ years), it is important to take in consideration early signs of cognitive decline that appear in midlife. This is because conditions such as dementia develop slowly and silently over the preceding decades [18–20]. Neuroimaging also show pathological changes in midlife before the clinical signs of the disease appear [21, 22]. Consistent with this, subtle cognitive decline begins as least as early as the 5th decade [23, 24].

Risk and protective factors for health can exert their most critical influences at different ages [25]. This is acknowledged by the life course approach and the hypothesis that positive lifestyle behaviors such as nonsmoking, being physically active, and choosing healthier diets may protect cognitive functioning and slow cognitive decline in later life. Fratiglioni et al. identified key periods for potential risk and protective factors [25]. Early life seems to be most critical for the development of cognitive reserve (learning and education) [26], when distal adverse influences (such as poor childhood social circumstances) contribute to the risk of adult disease or later life risk of dementia. Lifestyle behaviors, including those that influence cardiovascular and metabolic risk, become more influential in midlife, although some, such as diet and physical activity, track back into childhood [27, 28], whereas mental and physical activity patterns may continue to moderate these risks into later life [29, 30].

The fact that the lifestyle behaviors are modifiable implies that encouraging a healthy lifestyle may prevent or ameliorate cognitive decline and underlying cerebrovascular and cardiovascular risk factors [31]. Such interventions should take into account the relative beneficial effect of each independent behavior as well as their combined and cumulative effect.

### 1.1. Present Study

The aim of the current study was to examine the role of individual and combined lifestyle behaviors (smoking, physical activity, and dietary choice) on a 20-year interval of cognitive decline. We used measures of memory and psychomotor speed over this interval, which are sensitive to decline associated with ageing and neurodegeneration. The repeated measures of lifestyle behaviors at 36 and 43 years were used as predictors of cognitive change from 43 to 60–64 years. In addition to the independent and combined effects of these behaviors, we also examined the cumulative effects of these behaviors across early midlife and the changes in these behaviors from one age to another.

## 2. Methods

### 2.1. Study Members

**Figure 1:**
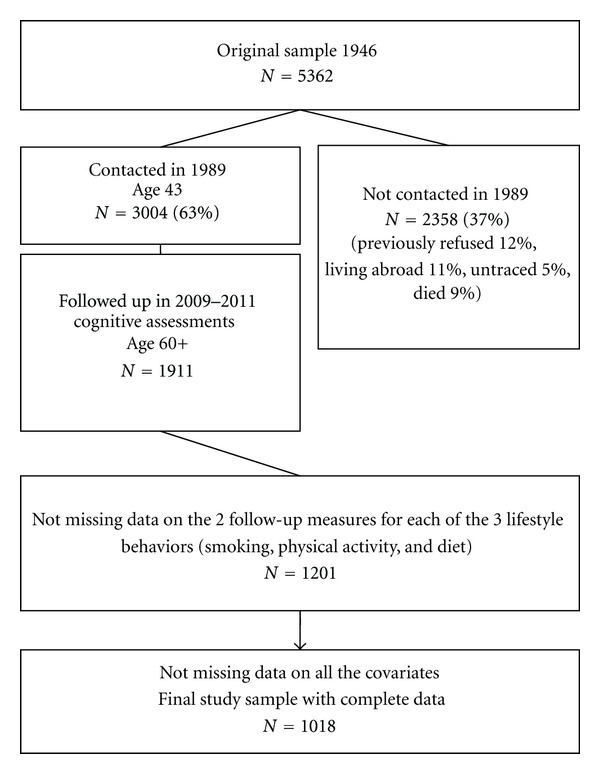
Flow chart of lifestyle behaviors, cognitive functioning and covariates data follow up (considered since baseline cognition-at age 43) in the Medical Research Council National Survey of Health and Development.

The Medical Research Council National Survey of Health and Development (NSHD) originally consisted of a socially stratified sample of 5362 children born within marriage in one week in March 1946 in England, Scotland, and Wales [32, 33]. This cohort has been followed up prospectively 23 times, from birth onwards. In 1989, when study members were aged 43 years, 3262 were successfully contacted, of whom 3004 have completed both cognitive tests. Of these, 1911 had successfully completed the most recent cognitive assessment, conducted between 2006 and 2011 when survey members were aged 60 to 64 years (henceforth 60+ years). This data collection began with a postal questionnaire sent to the target 3163 sample [33]. This was followed by an invitation to an assessment by trained nurses at one of six clinical research facilities (based in Cardiff, Birmingham, Edinburgh, London (at UCL and St Thomas' hospitals), and Manchester), or, if preferred, at a home visit. This was supplemented by a further questionnaire sent to those who agreed to a clinic or home visit. These questionnaires, along with additional questions asked by the nurses, updated information on general health, household composition, family structure, socioeconomic status, daily function, life events, and lifestyle. Contact was not attempted for 2198 (718 deaths, 567 living abroad, 594 prior refusals, and 320 permanently lost). From the current respondents, a total of 1,018 study members had nonmissing data for all variables incorporated in these analyses (see Figure 1).

### 2.2. Ethical Approval

The study protocol received ethical approval from the Greater Manchester Local Research Ethics Committee for the five English sites; from Scotland Research Ethics Committee for the data collection taking place in Edinburgh. Written informed consent was obtained from the study member at each stage of data collection.

### 2.3. Cognitive Function

Cognitive functioning was measured at 43 and 60+ years using two tests: verbal memory and speed and concentration. The verbal memory test consisted of a 15-item word learning task devised by the NSHD research team. Each word was shown for two seconds. When all 15 words were shown, the study member was asked to write down as many of these as possible, in any order. The total number of words correctly recalled over three identical trials was summed to provide an overall score for short-term verbal memory (maximum score = 45). This was followed by a letter search test (see below), after which an uncued delayed free recall trial was administered.

Speed and concentration were assessed with a letter search task in which participants were required to cross out the letters P and W, randomly embedded within a page of other letters, as quickly as possible within 1 minute. At age 43 years the total number of letters to be searched was 450, and at age 60+ years it was 600. Scores were computed as total number of letters searched.

### 2.4. Lifestyle Behaviors

Data on lifestyle behaviors were extracted from questionnaires, interview-based prospective information, and diet diaries completed in early midlife (36 and 43 years).

### 2.5. Smoking

Interview-based prospective information on cigarette smoking frequency was obtained at age 36 and 43 years. Smoking frequency was categorized at each age as 0 (nonsmoker), 1 to 20 (light smoker), or more than 20 cigarettes per day (heavy smokers).

A midlife smoking score was derived by assigning those classified as nonsmokers a value of 0, those as light smokers a value of 1, and those as heavy smokers a value of 2 at each age (36 and 43 years) and then summing the values for the 2 ages whereby an individual with a midlife smoking score of 0 was categorized as nonsmoker, an individual with a midlife smoking score of 1 to 3 was categorized as moderate smoker, and the remaining individuals were categorized as heavy smokers (sum equal 4).

A score for change in smoking behavior was also derived by assigning those classified as nonsmokers at each age a value of 0; those reporting an increase in smoking a score of 1; those who reported a decrease in smoking a score of 2; those who were constantly moderate or heavy smokers across early midlife a score of 3.

### 2.6. Physical Activity

Physical activity levels were ascertained at ages 36 and 43 years during interviews at the study participants' home. Questions about physical activity at age 36 years were based on the Minnesota leisure time physical activity questionnaire [27, 34]. The questions addressed engagement in sports and recreational activities in the previous month, utilizing a checklist of 27 different leisure time activities. At age 43 years, participation in any sports, vigorous leisure activities, or exercise was reported, although this was based on answers to an open-ended question, rather than the above checklist. However, the monthly frequency of these activities was also reported, enabling a similar categorization to the 36 year measure. At each age, participants were categorized as inactive (reported no participation); moderately active (participated in relevant activities 1–4 times in the previous month at age 36 years, and per month at age 43 years); or most active (participated in relevant activities five or more times in the previous month at age 36 years and per month at age 43 years) [35].

A total physical activity score was derived by assigning those classified as inactive a value of 0, those as moderately active a value of 1, and those as most active a value of 2 at each age (36 and 43 years) and then summing the values for the two ages. This was categorized as inactive (score 0), moderately active (score 1 to 3), and most active (score 4).

A score for change in physical activity behavior, was derived in a similar fashion as for smoking, by assigning those classified as inactive at each age a value of 0; those reporting an increase in activity a score of 1; those who reported a decrease in their physical activity levels a score of 2; those whose physical activity levels were moderate or most active at both ages a score of 3.

### 2.7. Dietary Choice

Dietary intake was assessed by a five-day diary [36] at both 36 and 43 years. All food and drinks consumed both at and away from home were recorded in the diaries, including brand names of food products, food preparation methods, and recipes used. Participants were asked to record the amount eaten in household measures, with guidance notes and photography provided in the diary to assist in estimating portion size [37]. From this information, an overall score representing level of healthy food choice was derived, by summing scores for four separate criteria: (1) consumption of breakfast (a score of 0 representing no consumption to 1 some days and 2 all days); (2) type of milk (from 0 whole only to 3 skim milk only); (3) type of bread (from 0 white only to 4 wholemeal only); (4) number of daily portions of fruit and vegetables (from 0 to maximum 5 portions per day); a dietary reference score representing the percentage of energy from fat, carbohydrates, and protein (scores from 1-highest to 5-lowest percentages less than 30% energy). The total score was subject to a median split (median = 10 at age 36 years and median = 11 at age 43 years; minimum score 0 and maximum 19) to represent low versus high (energy dense/nutrient poor versus healthier) dietary choice. The range of dietary scores varied from a minimum of 5 to a maximum of 19; *M* = 10.22, SD = 1.84 at age 36; *M* = 11.42, SD = 2.27 at age 43).

A midlife dietary choice score was also derived by assigning those classified as making poorer choices for their diet a value of 0 and those making healthier choices a value of 1 at each age (36 and 43 years) and then summing up the values for the 2 ages. Midlife dietary choice was categorized as making poorer (low) choices at both ages (score 0) and making healthier dietary choices (high) at least at one age (score 1 or 2).

A score for change in diet was also derived. Those classified as choosing a low-quality diet at each age were assigned a value of 0; those reporting an increase from a low-quality to a high-quality diet a score of 1; those who changed to a lower-quality diet a score of 2; those who constantly maintain a high-quality diet were assigned a score of 3.

### 2.8. Covariates

Based on previous findings, the following variables were treated as potential confounders: sex; father social class; childhood cognitive ability; educational attainment; midlife household occupational social class and depression [14, 38–40].

### 2.9. Occupational Social Class of Origin

*Occupational social class of origin* was represented by father's social class when participants were aged 11 years, or if this was unknown, at age 4 or 15 years. This was classified as professional managerial intermediate, skilled nonmanual, skilled manual, semi-skilled manual, or unskilled, according to the UK Registrar General's Classification of Occupations [41].

### 2.10. Prior Cognitive Ability

Childhood cognitive ability at age eight years was represented as the sum of four tests of verbal and nonverbal ability devised by the National Foundation for Educational Research [42]. These tests were (1) reading comprehension (selecting appropriate words to complete 35 sentences); (2) word reading (ability to read and pronounce 50 words); (3) vocabulary (ability to explain the meaning of 50 words); (4) picture intelligence, consisting of a 60-item nonverbal reasoning test.

### 2.11. Educational Attainment

The highest educational qualification achieved by age 26 years was dichotomized into those with advanced (“A level”, taken during the final year of secondary/high school) or higher (university or equivalent) qualifications, versus those below this level.

### 2.12. Occupational Social Class

Household midlife occupational social class was used at each time of the health behavior (36 or 43 years), or the latest age for longitudinal behavioral measures (see below). This was coded according to the Registrar General (as for social class of origin).

### 2.13. Depression and Anxiety Symptoms

Frequency and severity of common symptoms of depression and anxiety were assessed by the Psychiatric Symptom Frequency scale (PSF) [43] at age 43 years, and with the 28-item General Health Questionnaire (GHQ) [44] at age 60+.

### 2.14. Statistical Analyses

Multivariable ANCOVA models were used to test associations between smoking, physical activity, and diet at each age, entered as categorical variables and cognitive decline, used as continuous variables. First, we tested the association between each lifestyle behavior at 36 and 43 years and change in both cognitive outcomes (verbal memory and letter search speed); then we used the cumulative behavior scores for both these ages; finally we tested associations between change in behaviors between these ages and change in the cognitive outcomes between ages 43 and 60+ years. In order to reduce the effect of regression to the mean and because of the difference in size of the letter search matrix at ages 43 and 60+ years, we used conditional models of change by adjusting cognitive scores at age 60+ years for their corresponding score at 43 years [23]. Positive coefficients represent a slower rate of decline, and negative coefficients represented a faster rate of decline. Model 1 adjusted raw associations for sex (since there was no evidence of sex x lifestyle behavior interactions on the outcomes), social class of origin, prior cognitive ability at age 8 years, educational attainment, occupational class, and symptoms of anxiety and depression. Model 2 included these covariates but additionally tested the specificity of lifestyle behaviors by mutually adjusting each for the other behaviors. For cumulative effects, model 2 was further adjusted for the cumulative scores of the additional behaviour; for change in behaviour from 36 to 43, model 2 was mutually adjusted for the change in other lifestyle behaviours. All analyses were based on the sample with complete data on cognitive tests at the 2 time points, lifestyle behaviors at the 2 time points, and all the selected covariates.

## 3. Results

### 3.1. Sample Description and Missing Data

Participants with missing scores on memory and visual search at age 43 were more likely to be men, to have less than advanced educational attainment, to be in a manual occupation in midlife, and to have lower cognitive capability scores at age 8 years (all *P* < 0.001). A similar pattern was observed for those without cognitive scores at age 60+. Those with missing scores on memory at age 43 had a marginally higher total anxiety and depression score at the same age than those who underwent memory testing (*P* = 0.058). Those who did not complete data on lifestyle behaviors at both 36 and 43 years had lower cognitive scores for both verbal memory (*P* < 0.001) and visual search (*P* = 0.076; *P* = 0.010) at age 43 and 60+, were more likely to belong to a manual occupation of social class of origin (*P* < 0.001), and had less than advanced levels of educational attainment by age 26 years (*P* < 0.001).

Participants included in the current analysis were mostly nonsmokers at either 36 (76.6%) or 43 years (80.1%) and at least moderately active at ages 36 (68.1%), although less so at 43 years (53.2%), but had a similar quality of diet at both 36 and 43 years (55.4% and 51.1%, respectively, were in the lower median split).

Table 1 shows means for the total verbal memory and visual search speed scores at age 60+, by the three lifestyle behaviors (at 36 and 43 years, and the cumulative and change scores for these ages), by sex, father's social class, educational attainment, and midlife social class and depression of the 1018 participants. All three lifestyle behaviors (smoking, physical activity, and diet) at age 36, 43 years, as cumulative scores or change in behavior were strongly associated with verbal memory at age 60+. The effect was monotonic, with those who did not smoke or decreased their level of smoking; those who were most physically active or increased their level of activity or had a healthier or improved diet, having better memory scores at age 60+. For visual search speed at age 60+ the general trend was for slower function with heavy and increased smoking across early midlife; a positive association for high and increased physical activity; a positive association for consistently high and increased diet quality.

### 3.2. Association between Lifestyle Behaviors and Memory Decline

Table 2 shows results for the ANCOVAs for each health behavior (at 36 and 43 years, the cumulative and change scores for these ages) and memory decline from 43 to 60+ years. Trends can be observed for associations between heavy smoking at age 43 and for increase in smoking consumption and faster memory decline at all stages of model adjustment, but these trends were not significant at the *α* = 0.05 level. Directions of associations between physical activity and memory decline were inconsistent across the various categories of this behavior, but none were significant. On the other hand a consistently healthy dietary choice at 36 and 43 years was associated with slower memory decline, although none of the dietary change categories were associated with this outcome.

### 3.3. Association between Lifestyle Behaviors and Visual Search Speed Decline

Table 3 shows results for the ANCOVAs for each health behavior (at 36 and 43 years, and the cumulative and change scores for these ages) and decline in visual search speed from 43 to 60+ years. Heavy smoking at 43 years was associated with faster decline in search speed compared to nonsmokers after adjustment for the covariates in Model 1, but not at 36 years or at both these ages (the latter an effect of reduced statistic power) and not at 43 years after mutual adjustment for the other health behaviors. Nor were there associations between change in smoking and this outcome. On the other hand high physical activity at 43 years was associated with slower search speed decline, after full adjustment. This was also the case for those most active at both 36 and 43 years and for those who increased their level of activity between these ages. There were no associations at the *α* = 0.05 level between any measure of dietary choice and rate of search speed decline.

## 4. Discussion

The principal aim of this study was to test associations between lifestyle behaviors in early midlife and cognitive decline over 20 years. Key findings were that a consistently healthy dietary choice was associated with slower memory decline, and that consistently high or increasing physical activity from early midlife to midlife was associated with slower visual search speed decline, independently of each other lifestyle behavior and of social class of origin, childhood cognition, educational attainment, adult social class, and symptoms of anxiety and depression. Smoking was not associated with either cognitive outcome. It should be noted that the current findings for dietary choice and physical activity were not always consistent at different ages across midlife, compared to effects of the cumulative scores and change in behavior between the 2 time points. It will be important to seek replication elsewhere before these findings can be treated as authoritative.

### 4.1. Strengths and Limitations

Strengths of this study include use of a nationally representative sample; availability of a wide range of prospectively-obtained potential confounders, including childhood cognition to rule out selection by prior ability; a detailed assessment of lifestyle behaviors in midlife, including 5-day diet diaries; estimation of cumulative and change effects with two measures of health behaviors; availability of cognitive outcomes that are age and morbidity sensitive [45, 46]; a 20-year interval for capturing decline in these measures upto an age when decline may begin to have functional consequences. In addition, while there are many reports examining associations with single lifestyle behaviors [47–49], very little work has focused on the combined influence of these behaviors on cognitive functioning [10, 11] and none at all to our knowledge on cognitive decline. Furthermore, we believe that the long interval between repeat administrations of the cognitive tests would have minimized potential practice effects.

We should also highlight a number of limitations. First, there was a disproportional loss of those who were less advantaged, in terms of lower childhood cognitive ability, education, and SES, although we have no reason to suspect that this affected the pattern of results observed. Second, all information on lifestyle behaviors was dependent on self-report and was not validated by independent measures, for example, cotinine in the case of smoking. Third, there is the possibility of regression to the mean effect when analyzing cognitive ability data over a long period of time, for example, one may assume that some of those who had high cognitive ability score at age 43 may have lower scores at age 63 years and vice versa. We have tried to reduce this effect by using the conditional model of change [50].

 In regard to previous findings in NSHD, the inverse association between physical activity and memory decline from 43 to 53 years reported by Richards et al. [51] was not seen here for 20-year decline. This may be because the previous study had additional specificity through adjusting for non-physical spare-time activities, which would have been too cumbersome in the context of multiple lifestyle behaviors in the present study, or because of the longer period of cognitive change. The associations between physical activity and slower decline in visual search speed and between healthy dietary choice and memory are new findings and were not previously tested in this cohort; in the former case physical activity was not investigated in relation to search speed in the previous study; in the latter case midlife cognition has not previously been studied in relation to diet in this cohort. On the other hand the associations between heavy smoking at age 43 and faster memory decline previously reported between ages 43 and 53 years [40] were not replicated here with the 20-year period of cognitive change from 43 to 60+. The loss of the cumulative midlife heavy smoking-memory decline association may be due low statistical power resulting from the relatively high odds of morbidity and premature mortality in this subgroup (135 study members smoking more than 20 cigarettes per day at age 43 were represented in the previous study, compared to 22 in the present study).

In relation to other cohorts, Sabia et al. found an effect of sex on the association between smoking and cognitive decline in a study of London civil servants. Their results showed that men who smoked showed faster decline than nonsmoking men over a 10-year period, after adjusting for the effects of heart disease, stroke, and lung function on mental abilities, while for women there were no differences in cognitive scores over the same time period. This could be related to a lower number of female participants in contrast to males in the Whitehall II study [52]. In relation to physical activity, leisure-time physical activity at least twice a week in midlife was associated with reduced risk of memory decline in the Cardiovascular Risk Factors, Aging and Incidence of Dementia (CAIDE) study after adjustment for age, sex, education, follow-up time, locomotor disorders, APOE genotype, vascular disorders, smoking, and alcohol consumption [53]. Similarly, in The Mayo Clinic Study of Aging, moderate exercise in midlife or late life was associated with reduced odds of Mild Cognitive Impairment (MCI) [54]. In contrast, results from the Chicago Health and Aging Project reported that physical activity conducted within 2 weeks of the date of baseline cognitive assessment was not associated with risk of cognitive decline in an older population [55].

 Our finding that maintained healthy dietary choice was associated with a slower rate of memory decline is consistent with the results of a recent systematic review, which highlighted that a diet high in saturated fat represents an increased risk of cognitive decline and subsequent dementia [6]. The emphasis on identifying specific nutrients associated with cognitive ability in later life, such as antioxidants (vitamin C, E, carotenoids, and polyphenols), minerals, and dietary lipids (total, trans, and saturated mono-and polyunsaturated fats) [56], is now giving way to studies of global diet quality indices, for guidance in modeling dietary risk in relation to cognitive performance [57, 58]. In this context general recommendations are made for high fruit and vegetable consumption and moderation of high-glycemic index foods as cardioprotective, with secondary benefits to cognitive ageing [59]. The reason for the specificity of the diet memory association is unclear. Healthy dietary choice is protective of dementia [60], and evidence shows that dementia is predicted by memory impairment in particular [61, 62]. However, physical exercise, which was specifically associated with search speed, is also protective of dementia [63]. As already noted, however, the apparent loss of the exercise memory association reported by Richards et al. (2003) may be a consequence of not adjusting for non physical leisure activities here [51].

Our results for diet differ to those of The Nutrition et Cognition (NutCog) study, where diet quality was not independently associated with cognitive change over 3-year period. However, cognitive decline in this study was measured over a much shorter interval, although this study did report an association between diet quality and risk factors for nutrition-related chronic diseases, which are also considered to be risk factors for cognitive decline [56].

 There are several plausible biological mechanisms underlying associations between physical activity and cognitive decline. Physical activity reduces cardiovascular risk [64], increases cerebral perfusion, and facilitates neurogenesis [65–67]. In contrast, impaired blood flow in the midbrain [68, 69] is a risk factor for subsequent cognitive impairment and dementia. Of interest, physical activity was specifically associated with a slower decline in psychomotor speed, which is consistent with evidence that highly fit individuals respond faster to stimuli [70].

### 4.2. Conclusions and Implications

 In conclusion, our results support evidence that physical activity and healthy dietary choice are protective of aspects of cognitive ageing. These mutually adjusted associations were observed over a 20-year period, in analyses additionally controlling for socioeconomic status, sex, educational attainment, prior cognitive ability, and symptoms of anxiety and depression. Further work on interactions between lifestyle behaviors is recommended. For example, evidence suggests that smokers have poorer dietary choices than nonsmokers [71]; the Mediterranean diet only appears to be an effective protective factor for Alzheimer's disease in those who also exercise [72]. Overall, however, and in view of the enormous financial and societal burden of neurodegenerative diseases, public health interventions based on modifiable lifestyle behaviors across the life course represent high level priorities around the world.

## Figures and Tables

**Table 1:** Characteristics and mean scores for memory and search speed at age 60+ years, by lifestyle behaviour profiles and dichotomous covariates.

Variable	*N*	Memory at age 60+ years	*P*	Search speed at age 60+ years	*P*
Smoking at age:					
36 years					
Non-smoker	780	25.00 (5.88)	<0.001	273.47 (73.13)	0.002
1–20 c/day	200	24.18 (6.37)		266.7 (69.88)	
20+ cig/day	38	21.23 (7.33)		233.26 (59.33)	
43 years					
Non-smoker	816	25.05 (5.95)	<0.001	274.45 (72.55)	0.001
1–20 c/day	159	23.99 (6.05)		258.60 (68.67)	
20+ cig/day	43	20.72 (7.05)		242.83 (73.49)	
Midlife score:					
Non-smoker	744	25.05 (5.90)	<0.001	274.51 (73.69)	<0.001
Moderate smoker	252	24.07 (6.30)		263.48 (67.50)	
Heavy smoker	22	20.18 (7.25)		221.81 (59.74)	
Change in smoking:					
Non-smoker	744	25.05 (5.90)	0.005	274.51 (73.69)	0.011
Increase	55	22.89 (5.91)		254.63 (65.05)	
Decrease	79	24.77 (6.51)		272.29 (60.10)	
Constant med/heavy	140	23.52 (6.60)		255.44 (72.33)	
Physical activity at age:					
36 years					
Inactive	324	23.82 (5.83)	<0.001	264.87 (72.81)	0.138
Moderate active	275	24.61 (5.98)		270.04 (68.14)	
Most active	419	25.44 (6.24)		275.49 (74.58)	
43 years					
Inactive	475	23.85 (6.13)	<0.001	263.83 (69.38)	<0.001
Moderate active	251	25.15 (6.05)		267.10 (69.13)	
Most active	292	25.69 (5.84)		284.76 (78.01)	
Midlife score:					
Inactive	221	23.37 (6.01)	<0.001	259.08 (74.61)	0.003
Moderate active	608	24.81 (5.98)		270.87 (68.78)	
Most active	189	25.90 (6.20)		283.43 (79.00)	
Change in physical activity:					
Inactive	221	23.37 (6.01)	<0.001	259.08 (74.61)	0.003
Increase	164	25.03 (5.35)		282.39 (74.40)	
Decrease	354	24.61 (6.30)		266.84 (66.91)	
Constant active	279	25.68 (6.08)		277.72 (74.71)	
Diet at age:					
36 years					
Low quality	564	24.01 (6.19)	<0.001	267.26 (69.12)	0.096
High quality	454	25.55 (5.84)		274.85 (76.14)	
43 years					
Low quality	521	23.72 (6.13)	<0.001	267.24 (69.30)	0.124
High quality	497	25.72 (5.86)		274.21 (75.41)	
Midlife score:					
Low quality	234	22.51 (5.90)	<0.001	260.02 (67.96)	0.010
High quality	784	25.34 (5.99)		273.81 (73.41)	
Change in diet:					
Constant low quality	234	22.55 (5.90)	<0.001	260.02 (67.96)	0.022
Increase	330	25.06 (6.18)		272.39 (69.57)	
Decrease	94	23.98 (5.44)		263.80 (71.44)	
Constant high	360	25.96 (5.88)		277.73 (77.16)	
Sex:					
Male	490	23.72 (5.94)	<0.001	265.36 (71.84)	0.024
Female	528	25.61 (6.07)		275.54 (72.63)	
Father social class					
Non-manual	507	26.28 (5.90)	<0.001	278.69 (75.64)	<0.001
Manual	511	23.12 (5.84)		262.66 (68.16)	
Education:					
<advanced	567	22.77 (5.66)	<0.001	263.03 (70.09)	<0.001
≥advanced	451	27.12 (5.71)		280.21 (74.17)	
Midlife social class at age:					
36 years					
Non-manual	617	26.24 (5.79)	<0.001	280.36 (71.29)	<0.001
Manual	401	22.33 (5.75)		255.69 (71.62)	
43 years					
Non-manual	696	26.08 (5.79)	<0.001	276.31 (71.73)	<0.001
Manual	322	21.72 (5.60)		258.38 (72.42)	
Depression at age:					
43 years					
No symptoms	966	24.80 (6.05)	0.036	271.08 (72.92)	0.403
Depressive symptoms	52	22.98 (6.47)		262.46 (61.78)	
60+ years					
No symptoms	805	24.85 (6.09)	0.194	270.03 (73.61)	0.531
Depressive symptoms	171	24.19 (5.74)		273.87 (69.23)	

**Table 2:** Regression coefficients (95% confidence intervals) representing rate of decline in verbal memory from 43 to 60+ years per level increase in each health behavior at 36 and 43 years and per cumulative and change in health behavior scores.

	*N* (%)	Verbal memory decline 43 to 60+ (95% CI)	
		Model 1	Model 2
Smoking at age:			
36 y			
Non-smoking	780 (76.6)	0.00	0.00
1–20 c/day	200 (19.6)	−0.16 (−0.85, 0.53)	−0.12 (−0.81, 0.57)
20+ cig/day	38 (3.7)	−0.22 (−1.67, 1.22)	−0.17 (−1.62, 1.28)
*P* value		0.610	0.701
43 y			
Non-smoking	816 (80.1)	0.00	0.00
1–20 c/day	159 (15.6)	−0.08 (−0.83, 0.67)	−0.07 (−0.83, 0.69)
20+ cig/day	43 (4.2)	−1.38 (−2.75, −0.01)	−1.24 (−2.61, 0.11)
*P* value		0.124	0.165
Midlife score:			
Non-smoker	744 (73.0)	0.00	0.00
Moderate smoker	252 (24.7)	−0.20 (−0.83, 0.43)	−0.12 (−0.76, 0.51)
Heavy smokers	22 (2.1)	−0.22 (−2.10, 1.66)	−0.01 (−1.90, 1.87)
*P* value		0.535	0.750
Change in smoking:			
Non-smoker	744 (73.0)	0.00	0.00
Increase	55 (5.4)	−1.11 (−2.32, 0.09)	−1.00 (−2.21, 0.21)
Decrease	79 (7.7)	0.10 (−0.91, 1.12)	0.09 (−0.92, 1.11)
Constant med/heavy	140 (13.7)	−0.03 (−0.83, 0.77)	0.04 (−0.78, 0.86)
*P* value		0.363	0.473
Physical activity at age:			
36 y			
Inactive	324 (31.8)	0.00	0.00
Moderately active	275 (27.0)	−0.06 (−0.76, 0.64)	−0.07 (−0.78, 0.63)
Most active	419 (41.1)	0.21 (−0.42, 0.86)	0.17 (−0.47, 0.82)
*P* value		0.484	0.571
43 y			
Inactive	475 (46.6)	0.00	0.00
Moderately active	251 (24.6)	0.06 (−0.61, 0.75)	0.02 (−0.65, 0.71)
Most active	292 (28.6)	−0.06 (−0.72, 0.59)	−0.12 (−0.79, 0.54)
*P* value		0.878	0.731
Midlife score:			
Inactive	221 (21.7)	0.00	0.00
Moderate	608 (59.7)	−0.13 (−0.82, 0.54)	−0.23 (−0.91, 0.45)
Most active	189 (18.5)	−0.16 (−1.05, 0.71)	−0.31 (−1.20, 0.56)
*P* value		0.697	0.472
Change in activity:			
Inactive	221 (21.7)	0.00	0.00
Increase	164 (16.1)	−0.35 (−1.25, 0.54)	−0.47 (−1.37, 0.43)
Decrease	354 (34.7)	−0.10 (−0.84, 0.63)	−0.18 (−0.92, 0.55)
Constant mod/active	279 (27.4)	−0.06 (−0.87, 0.73)	−0.17 (−0.98, 0.63)
*P* value		0.871	0.768
Diet at age:			
36 y			
Low-quality diet	564 (55.4)	0.00	0.00
High-quality diet	454 (44.6)	0.38 (−0.17, 0.93)	0.35 (−0.20, 0.91)
*P* value		0.176	0.212
43 y			
Low-quality diet	521 (51.1)	0.00	0.00
High-quality diet	497 (48.8)	0.20 (−0.34, 0.75)	0.17 (−0.38, 0.73)
*P* value		0.468	0.549
Midlife score:			
Low-quality diet	234 (22.9)	0.00	0.00
High-quality diet	784 (77.0)	0.71 (0.04, 1.38)	0.70 (0.02, 1.38)
*P* value		0.037	0.043
Change in diet:			
Low-quality	234 (22.9)	0.00	0.00
Increase in quality	330 (32.4)	0.65 (−0.08, 1.40)	0.65 (−0.10, 1.41)
Decrease in quality	94 (9.2)	0.95 (−0.09, 2.00)	0.91 (−0.14, 1.97)
Constant high-quality	360 (35.3)	0.78 (0.02, 1.53)	0.76 (0.00, 1.54)
*P* value		0.146	0.180

Model 1: model adjusted for sex, childhood social class, childhood cognition at age 8, SEP (own occupation and education), and depression.

Model 2: model 1 plus other lifestyle behaviors.

**Table 3:** Regression coefficients (95% confidence intervals) representing rate of decline in visual search from 43 to 60+ years per level increase in each health behavior at 36 and 43 years and per cumulative and change in health behavior scores.

	*N* (%)	Visual search decline 43 to 60+ (95% CI)	
		Model 1	Model 2
Smoking at age:			
36 y			
Non-smoking	780 (76.6)	0.00	0.00
1–20 c/day	200 (19.6)	−1.90 (−11.77, 7.96)	−1.79 (−11.68, 8.09)
20+ cig/day	38 (3.7)	−18.70 (−39.27, 1.86)	−19.44 (−40.05, 1.17)
*P* value		0.156	0.148
43 y			
Non-smoking	816 (80.1)	0.00	0.00
1–20 c/day	159 (15.6)	−4.19 (−14.92, 6.53)	−2.73 (−13.55, 8.08)
20+ cig/day	43 (4.2)	−19.54 (−38.97, −0.12)	−18.52 (−38.00, 0.94)
*P* value		0.060	0.103
Midlife score:			
Non-smoker	744 (73.0)	0.00	0.00
Moderate smoker	252 (24.7)	−4.85 (−13.93, 4.21)	−4.82 (−13.93, 4.28)
Heavy smokers	22 (2.1)	−24.80 (−51.61, 2.00)	−25.46 (−52.34, 1.42)
*P* value		0.078	0.077
Change in smoking:			
Non-smoker	744 (73.0)	0.00	0.00
Increase	55 (5.4)	−13.08 (−30.17, 4.00)	−12.78 (−30.02, 4.45)
Decrease	79 (7.7)	−2.70 (−17.06, 11.64)	−2.65 (−17.09, 11.78)
Constant med/heavy	140 (13.7)	−6.71 (−18.09, 4.67)	−5.82 (−17.44, 5.79)
*P* value		0.374	0.455
Physical activity at age:			
36 y			
Inactive	324 (31.8)	0.00	0.00
Moderately active	275 (27.0)	2.69 (−7.29, 12.68)	3.20 (−6.80, 13.20)
Most active	419 (41.1)	8.00 (−1.12, 17.13)	8.47 (−0.70, 17.65)
*P* value		0.081	0.067
43 y			
Inactive	475 (46.6)	0.00	0.00
Moderately active	251 (24.6)	−3.93 (−13.57, 5.69)	−4.37 (−14.03, 5.28)
Most active	292 (28.6)	11.84 (2.55, 21.12)	11.18 (1.78, 20.58)
*P* value		0.022	0.034
Midlife score:			
Inactive	221 (21.7)	0.00	0.00
Moderate	608 (59.7)	8.32 (−1.33, 17.97)	8.76 (−0.95, 18.47)
Most active	189 (18.5)	16.71 (4.32, 29.10)	17.00 (4.50, 29.51)
*P* value		0.008	0.008
Change in activity:			
Inactive	221 (21.7)	0.00	0.00
Increase	164 (16.1)	14.45 (1.83, 27.07)	13.78 (1.05, 26.50)
Decrease	354 (34.7)	7.58 (−2.87, 18.03)	7.80 (−2.72, 18.34)
Constant mod/active	279 (27.4)	10.51 (−0.74, 21.77)	10.46 (−0.92, 21.84)
*P* value		0.127	0.157
Diet at age:			
36 y			
Low-quality diet	564 (55.4)	0.00	0.00
High-quality diet	454 (44.6)	−0.52 (−8.39, 7.34)	−1.74 (−9.67, 6.19)
*P* value		0.896	0.667
43 y			
Low-quality diet	521 (51.1)	0.00	0.00
High-quality diet	497 (48.8)	0.59 (−7.23, 8.42)	−1.03 (−8.91, 6.84)
*P* value		0.881	0.797
Midlife score:			
Low-quality diet	234 (22.9)	0.00	0.00
High-quality diet	784 (77.0)	1.59 (−7.89, 11.09)	−0.64 (−10.28, 9.00)
*P* value		0.741	0.896
Change in diet:			
Low-quality	234 (22.9)	0.00	0.00
Increase in quality	330 (32.4)	1.53 (−9.04, 12.12)	−0.69 (−11.45, 10.06)
Decrease in quality	94 (9.2)	−1.23 (−16.11, 13.64)	−3.22 (−18.18, 11.74)
Constant high-quality	360 (35.3)	1.63 (−9.09, 12.36)	−1.39 (−12.31, 9.57)
*P* value		0.972	0.978

Model 1: model adjusted for sex, childhood social class, childhood cognition at age 8, SEP (own occupation and education), and depression.

Model 2: model 1 plus other lifestyle behaviors.
